# The “Reducing Inflammation for Greater Health Trial (RIGHT)” Study—Concept, Rationale, and Design

**DOI:** 10.1111/jgs.70272

**Published:** 2026-01-10

**Authors:** Sebastian E. Sattui, Marnie Bertolet, Daniel E. Forman, Michelle E. Danielson, Shanshan Yao, Oscar L. Lopez, Nancy W. Glynn, Neelesh K. Nadkarni, Akira Sekikawa, Tullia C. Bruno, Toren Finkel, Anne B. Newman

**Affiliations:** ^1^ Department of Medicine, School of Medicine University of Pittsburgh Pittsburgh Pennsylvania USA; ^2^ Department of Epidemiology, School of Public Health University of Pittsburgh Pittsburgh Pennsylvania USA; ^3^ Geriatrics Research Education and Clinical Care (GRECC) VA Pittsburgh Healthcare System Pittsburgh Pennsylvania USA; ^4^ Aging Institute of the University of Pittsburgh/UPMC Pittsburgh Pennsylvania USA; ^5^ Department of Neurology, School of Medicine University of Pittsburgh Pittsburgh Pennsylvania USA

**Keywords:** aging, clinical trial, inflammation

## Abstract

The Reducing Inflammation for Greater Health Trial's (RIGHT) study is a single‐center, randomized, double‐blind, placebo‐controlled trial designed to test whether clazkizumab, an interleukin‐6 (IL‐6) inhibitor, can improve or slow decline in physical, cognitive, and vascular function in older adults, when compared to a placebo. The trial will enroll participants meeting the following inclusion criteria: (1) ≥ 70 years of age, (2) with low to moderate physical function, defined as self‐reported difficulty walking 1/4 mile or climbing a flight of stairs, but able to walk 400 m at baseline exam, (3) usual walking speed between ≥ 0.44 and < 1.0 m/s on a 4‐m walk or a body mass index of ≥ 28 kg/m^2^, (4) average IL‐6 level between 2.0 and 30 pg/mL on two tests, and (5) no active infection, cancer, or other serious health conditions. Clazakizumab, a monoclonal antibody targeting IL‐6, 5 mg via subcutaneous injection every 4 weeks for 24 weeks compared to a placebo. The primary outcome will be walking speed over 400 m. Secondary outcomes include other measures of physical function (short physical performance battery, oxygen consumption with walking on a treadmill, fatigability), cognitive function, vascular stiffness and endothelial function, IL‐6 and C‐reactive protein levels, other markers of inflammation, safety, and tolerability. Findings will evaluate acceptability, safety and 6‐months change in mobility and other outcomes. The study was approved by the IRB and is registered with ClinicalTrials.gov (NCT05727384). The RIGHT study will inform the geroscience hypothesis that modifying aging itself will lead to improvement in multiple aspects of health.

## Summary

- Key points
  ○ Clinical trials studying therapies that can maintain or improve function as people age are essential given the growing population of older adults.
  ○ Assessing the effect of the direct interleukin‐6 inhibition on age‐related decline could inform the geroscience hypothesis that modifying aging itself can improve multiple aspects of health.
  ○ The RIGHT trial will assess the feasibility, acceptability, safety, and tolerability of clazakizumab compared to placebo and estimate its effect on walking speed.
- Why does this paper matter?
  ○ This manuscript describes a novel geroscience trial, Reducing Inflammation for Greater Health Trial (RIGHT), a randomized placebo‐controlled trial studying the impact of an interleukin‐6 inhibitor on walking speed and other age‐related outcomes.

## 1 Introduction

The growing population of older adults in the United States underscores the urgent need for therapies that maintain or improve function as people age [1]. Biological aging is the primary driver of functional decline, even in healthy older adults [2]. One hallmark of aging is chronic low‐grade inflammation, or “inflammaging,” characterized by elevated inflammatory markers such as C‐reactive protein (CRP), tumor necrosis factor‐α (TNF‐α), and interleukin‐6 (IL‐6) [3]. Epidemiologic cohort studies, including the Health, Aging and Body Composition (Health ABC) Study, the Framingham Heart Study, and the Cardiovascular Health Study [4, 5], have demonstrated robust links between these inflammatory markers and age‐related health outcomes [6], including cardiovascular disease, sarcopenia, cognitive decline, and fractures [7, 8, 9, 10, 11, 12, 13]. IL‐6 is the strongest predictor [9, 10] of these outcomes, making it a promising surrogate and therapeutic target.

Evidence from animal studies supports the role of inflammation in modulating both lifespan and healthspan. A recent study showed that IL‐11 is upregulated across multiple cell types and tissues in aging mice. Deleting IL‐11 or administering an anti‐IL‐11 antibody to 75‐week‐old mice for 25 weeks protected them against metabolic decline, multimorbidity, and frailty while extending their lifespan [14]. These findings suggest that targeting IL‐11 influences the ERK‐AMPK‐mTORC1 axis, potentially modulating aging processes at multiple biological levels. Notably, anti‐IL‐11 is already under investigation in early‐stage clinical trials for fibrotic lung disease [15]. Since IL‐11 is part of the IL‐6 family, this data highlights the potential role of IL‐6 inhibition in modulating aging processes.

Inflammation is also a target for reducing cardiovascular disease risk. The Canakinumab Anti‐Inflammatory Thrombosis Outcomes Study (CANTOS) demonstrated that IL‐1β inhibition with canakinumab reduced recurrent cardiovascular disease in patients with a history of myocardial infarction [16]. It also showed a notable reduction of aging‐related disorders including lung cancer, highlighting potential wide‐ranging benefits beyond cardiovascular disease [17]. Subsequent analysis suggested these effects were mediated by IL‐6 reduction. To target IL‐6 directly, the RESCUE trial utilized ziltivekimab, an IL‐6 ligand‐targeting monoclonal antibody, to reduce cardiovascular risk in patients with advanced kidney disease and elevated CRP [18]. Building on these studies, we hypothesize that therapies directly targeting IL‐6 could be beneficial in addressing broader aging outcomes, including multimorbidity and age‐related functional decline.

Previously, the Enabling Reduction of Low‐Grade Inflammation in Seniors (ENRGISE) trial [19, 20] used safer agents like fish oil or losartan but did not achieve IL‐6 reduction. However, it demonstrated the feasibility of identifying, recruiting, and enrolling older adults at high risk of mobility disability—those most likely to benefit from interventions and most likely to experience measurable outcomes within a trial period [21, 22]. Informed by these findings, the Reducing Inflammation for Greater Health (RIGHT) study uses clazakizumab, a high‐affinity humanized monoclonal antibody directly targeting IL‐6 to address key geroscience outcomes. In this report, we describe the protocol, recruitment strategies, outcomes, and safety assessments.

## 2 Methods

### 2.1 Study Overview and Setting

This is a randomized, double‐blind, parallel, placebo‐controlled, pilot study in adults ≥ 70 years old, designed to assess the feasibility, acceptability, safety and tolerability of 5 mg clazakizumab via subcutaneous injection every 4 weeks for 24 weeks compared to a placebo injection, and to estimate its effects on physical function, brain health, vascular function, and inflammatory biomarkers. The RIGHT study is a single site study conducted at the University of Pittsburgh.

**FIGURE 1:**
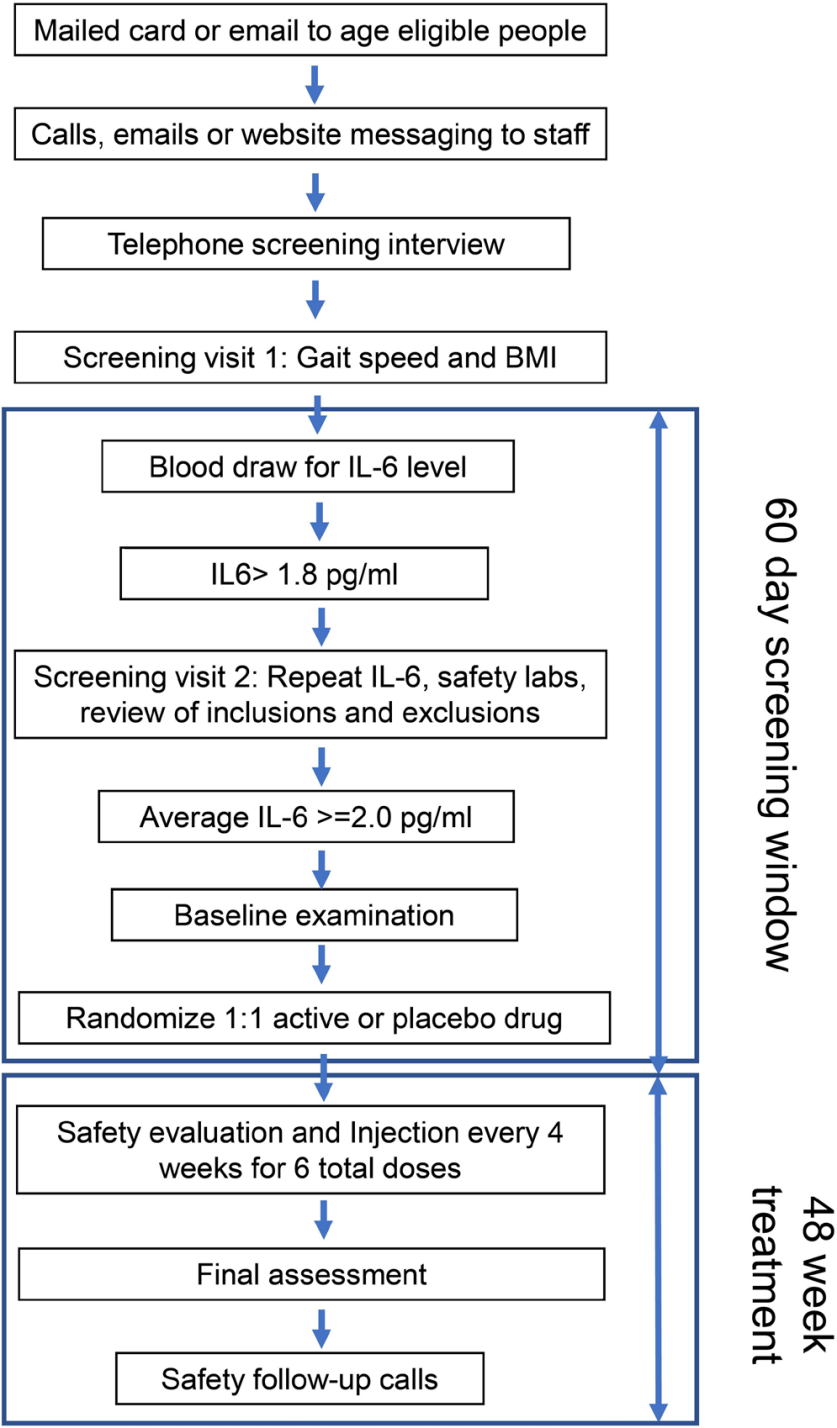
The RIGHT study visit flow chart. BMI = body mass index; IL‐6 = Interleukin‐6.

Following a telephone screening interview, candidates are seen for a first screening visit to obtain screening informed consent and to assess walking ability, body mass index (BMI), and IL‐6 level (Figure 1 and Table 1). If their usual pace walking speed in the 4‐m walking test is between ≥ 0.44 and < 1.0 m per second (m/s) or BMI ≥ 28 kg/m^2^, a first IL‐6 level is drawn. Given that previous studies have shown that older adults able to complete 400 m (primary outcome) without sitting down usually achieve this in under 15 min, we established a lower limit speed criterion to screen out those unable to do so (i.e., minimum speed of 0.44 m/s). If qualified after the screening visit, a more comprehensive second screening visit is conducted with a detailed medical history and medication review, repeat IL‐6 level, and blood work. If the average of two IL‐6 levels is ≥ 2.0 but < 30 pg/mL, and no exclusion criteria are identified (Table 2), a full study informed consent followed by a comprehensive baseline assessment is performed, including a 400‐m walk test as well as secondary and exploratory outcomes.

**TABLE 1:** The RIGHT study schedule.

Visit type	Telephone screening	Screening visit 1	Screening visit 2	Baseline visit/Drug administration visit 1	Safety/Drug administration visits 2–6	Safety/phone calls	6‐month follow‐up (final research assessment visit)	Follow‐up calls
*Measurement*								
Basic eligibility screening	x							
Short screening consent		x						
4‐m walk test		x	x^a^					
IL‐6 blood tests (eligibility screening)		x	x					
Medical history and medications review			x	x	x	x	x	x
Chest X‐ray			x					
Fasting blood collection for eligibility screening and safety tests			x		x		x	
Vital signs		x^b^	x	x	x		x	
Main informed consent				x				
Physical exam				x	x			
Anthropometric measures		x^c^		x			x	
Research blood				x			x	
Systematic adverse event assessments					x	x	x	x
Questionnaires (sleep, depression, functional status, fatigability)				x	x		x	
Cognitive testing (MoCA, DSST, Trails B, CVLT)				x			x	
400 m, walk with accelerometry, preferred and slow walking speed (with oxygen utilization) walk, SPPB, grip strength				x			x	
Accelerometry			x				x	
Aortic pulse wave velocity and endothelial responses				x			x	
Spirometry				x			x	
Brain MRI				x			x	
Addendum informed consent				x				
Administer study medication				x	x			
Proxy interview (if needed)							x	x
Blood drawn per visit		15	45	75	15 × 15		75	
Total = 285 mL		15	45	75	75		75	

Abbreviations: CVLT = California Verbal Learning Test; DSST = Digit Symbol Substitution Test; IL‐6 = interleukin‐6; MoCA = Montreal Cognitive Assessment; MRI = magnetic resonance imaging; SPPB = Short Physical Performance Battery; Trails B = Trail Making Test Part B.

^a^ If not done at SV1.

^b^ Temperature only at SV1.

^c^ Height and weight only.

**TABLE 2:** The RIGHT study inclusion and exclusion criteria.

Inclusion criteria
Persons aged ≥ 70 years at time of randomization
Gait speed ≥ 0.44 to < 1.0 m/s or BMI ≥ 28 kg/m^2^
Average IL‐6 level measured at SV1 and SV2 ≥ 2.0 pg/mL but < 30.0 pg/mL
Self‐reported difficulty walking 1/4 mile or climbing 10 steps
Self‐reported ability to walk 400 m (about 2–3 blocks) unassisted
Able to complete the 400 m walk test within 15 min without sitting or the help of another person and without a walker. A cane is allowed.
Self‐reported vaccinations for COVID‐19, Influenza and pneumococcal pneumonia up‐to‐date per current CDC guidelines
Exclusion criteria
Prevalent disability and disability risk:
Advanced neurologic disorder such as dementia, Parkinson's disease, amytrophic lateral sclerosis, or multiple sclerosis that would impact the ability to improve on functional assessments
Resident in a nursing home
Severe hearing or vision loss that would impair participant's ability to complete questionnaires or follow oral instructions, and which may limit feasibility of functional assessments
Infections, autoimmune disease or impaired immune function:
Acute infections (including but not limited to common cold virus, shingles virus, bronchitis, skin infection, urinary tract infection, tooth abscess) within 60 days of randomization
Chronic infection (including but not limited to):
History of active TB or evidence of latent TB based on a positive PPD skin test, positive Quantiferon TB‐Gold test or a history of old or latent TB on chest X‐ray
History of Hepatitis B or Hepatitis C
Previous diagnosis of Human Immunodeficiency Virus (HIV) or Acquired ImmunoDeficiency Syndrome (AIDS)
Inflammatory or autoimmune disease (including but not limited to rheumatoid arthritis, lupus, or inflammatory bowel disease, such as ulcerative colitis or Crohn's disease)
Immunization with a live/attenuated vaccine within 2 months prior to randomization (e.g., viral: measles vaccine, mumps vaccine, rubella vaccine, live attenuated influenza vaccine, live attenuated chicken pox or shingles vaccine, smallpox vaccine, oral polio vaccine [Sabin], rotavirus vaccine, and yellow fever vaccine. Bacterial: BCG vaccine, oral typhoid vaccine and epidemic typhus vaccine)
Current use of chronic immune modulating medications such as corticosteroids, monoclonal antibodies, Janus kinase inhibitors, calcineurin inhibitors, mTOR inhibitors, IMDH inhibitors, or biologics
Advanced or unstable medical conditions:
Admitted for an overnight hospital hospitalization in the last 6 months
Open‐chest heart surgery (including, but not limited to coronary artery bypass graft surgery or aortic valve surgery) in the past 6 months
Anticipating major surgery (including but not limited to chest, abdomen or joint surgery) in the next 6 months
Deep vein thrombosis or pulmonary embolus in the past 6 months
Severe lung disease or heart disease that requires oxygen use anytime during the day (including, but not limited to, use only during activity, use only at night, or use all day)
Tobacco use (including cigarettes, cigar, pipe, or vaping) or inhaled cannabis in the past 6 months
Current consumption of > 14 alcoholic drinks per week
History of substance abuse including cocaine, methamphetamine, opioids, or narcotics; any use of cannabis
Uncontrolled diabetes, non‐compliant with treatment or fasting glucose > 250 mg/dL
Cancer: Stage 1 cancer (including melanoma skin cancers) within the past 5 years, other than adequately treated (fully excised and recovered from surgery based on the judgment of a study MD) basal and/or squamous cell skin cancer, or stage 2 or stage 3 cancer within 10 years, or any history of stage 4 (metastatic) cancer
Inability to get a normal systolic blood pressure reading (between 100 and 180) at two consecutive visits prior to randomization (must be at least 1 day apart)
ALT, AST, or Total Bilirubin > ULN
Absolute Neutrophil Count outside normal range, or < 1.5 × 10^9^/L
White Blood Count outside normal range
Platelet count outside normal range, or < 125 × 10^9^/L
Hemoglobin < 10 g/dL
Total cholesterol > 300 mg/dL or Triglycerides > 400 mg/dL
Dialysis treatment or chronic renal insufficiency defined as CKD‐EPI eGFR < 25 mL/min/(1.73) m^2^
History of diverticular disease or GI perforation
Drug allergy or drug interactions:
History of severe allergic or anaphylactic reactions to human, humanized, or murine monoclonal antibodies
Current use of Warfarin
Factors that affect feasibility of safely completing study requirements:
Unable or unwilling to provide informed consent
Current participation in another interventional study (including trials of exercise, diet, or investigational drugs)
A psychiatric disorder that is impairing ability to consent or comply with requirements of the trial
Residence or travel outside of the study area for more than1month during the study or planning to move out of the area in the next 6 months
Other conditions which at the discretion of a study physician investigator which would make participation unsafe or inappropriate (logistic, behavioral, and medical)

Abbreviations: ALT = alanine aminotransferase; AST = aspartate aminotransferase; BMI = body mass index; CDC = Centers for Disease Control; CKD‐EPI eGFR = chronic kidney disease epidemiology collaboration estimated glomerular filtration rate; IMDH = inosine monophosphate dehydrogenase inhibitors; mTOR = mammalian target of rapamycin; PPD = purified protein derivative; SV = study visit; TB = tuberculosis; ULN = upper limit normal.

Randomization to study drug or placebo occurred within 60 days from the first IL‐6 screen. A study physician obtains additional consent prior for study drug injection just before randomization. All personnel assessing post‐randomization measurements and participants are masked to treatment assignment. Injections begin on the day of randomization and continue every 4 weeks (six total), provided that there are no safety issues in health history, exam or laboratory tests. After the final injection, a second comprehensive exam, same as the baseline exam, is conducted. Interim safety calls are conducted weekly for the first 4 weeks and then 1 week after each injection. After the completion of the injections, safety calls occur every 4 weeks for 20 weeks. Clazakizumab is provided by CSL Behring and stored at the University of Pittsburgh Investigational Drug Services pharmacy. The study protocol has been approved by the University of Pittsburgh Institutional Review Board and is monitored by a Data Safety Monitoring Board (DSMB). The study is registered with clinicaltrials.gov (NCT05727384).

### 2.2 Target Population and Recruitment

The target population is community‐dwelling older adults ≥ 70 years in stable health. Since the target population is a community population, recruitment efforts included mass mailings and emails using University of Pittsburgh research registries and purchased mailing lists of age‐eligible individuals. Flyers, postcards, and web‐based media describing the study and eligibility criteria were designed. Interested individuals call or email the study staff for a telephone screen for preliminary assessment of inclusion and exclusion criteria. People reporting any difficulty walking 1/4 mile and no major exclusions are invited for an in‐person exam.

### 2.3 IL‐6 Measurement

Weekly batches of IL‐6 screens are conducted at the University of Pittsburgh Immunologic Monitoring and Cellular Products Laboratory (IMCPL) of the Hillman Cancer Center using the Mesoscale Diagnostics V‐PLEX PLUS Human IL‐6 Kit platform that is sensitive to low levels of IL‐6. If the initial IL‐6 level is ≥ 1.8 pg/mL but < 30 pg/mL, a second IL‐6 level is measured for each study candidate. This two‐step process accounts for measurement variability based on regression to the mean, addressing the deviation of the initial value from the individual's true value.

**FIGURE 2:**
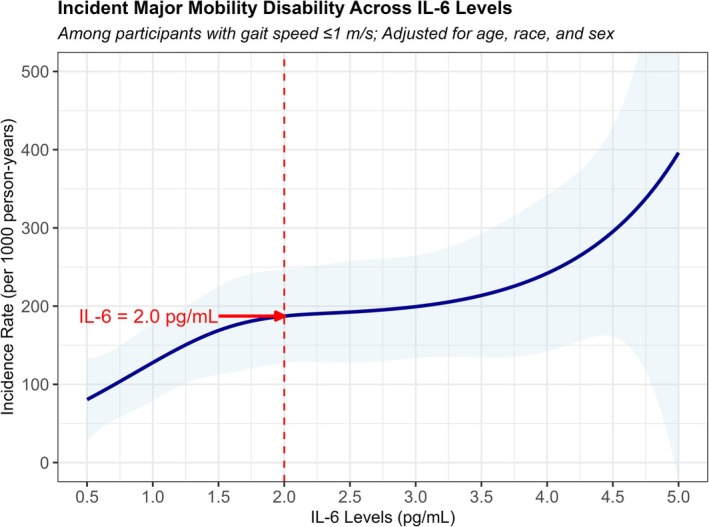
Incident major mobility disability in Health ABC study participants according to baseline IL‐6 level. Health ABC study = Health, Aging and Body Composition Study; IL‐6 = interleukin‐6.

The initial criterion for enrollment in RIGHT for IL‐6 was 2.5 pg/mL based on the ENRGISE trial [19]. Based on a reanalysis of Health ABC data, the lower limit was changed to an average IL‐6 of 2.0 pg/mL. In that analysis, a subsample was selected for similar eligibility criteria to the RIGHT trial, including age ≥ 70 years, gait speed ≤ 1.0 m/s, and no major mobility disability at baseline. The incidence of major mobility disability was found to be elevated above IL‐6 values of 1.0 pg/mL but seemed to peak around 2.0 pg/mL (Figure 2). We also examined early screening data from RIGHT and found that a value of 2.0 pg/mL was approximately the upper quartile of values for early screenees from their first screening visit. On this basis, we changed the criteria to use an average IL‐6 ≥ 2.0 and < 30 pg/mL for eligibility.

### 2.4 Inclusion and Exclusion Criteria

Criteria were developed from the ENRGISE Trial as well as trials using IL‐6 inhibitors in conjunction with CSL Behring medical safety staff (Table 2). Exclusion criteria are designed to exclude those with or at high risk of infections or severe uncontrolled medical conditions and individuals at higher risk of specific adverse events (AEs) based on the known safety profile of clazakizumab. As commonly seen in trials including older adults, high rates of multimorbidity and polypharmacy are expected in spite of fairly extensive exclusion criteria, especially as higher IL‐6 levels are associated with multimorbidity.

### 2.5 Study Drug

**FIGURE 3:**
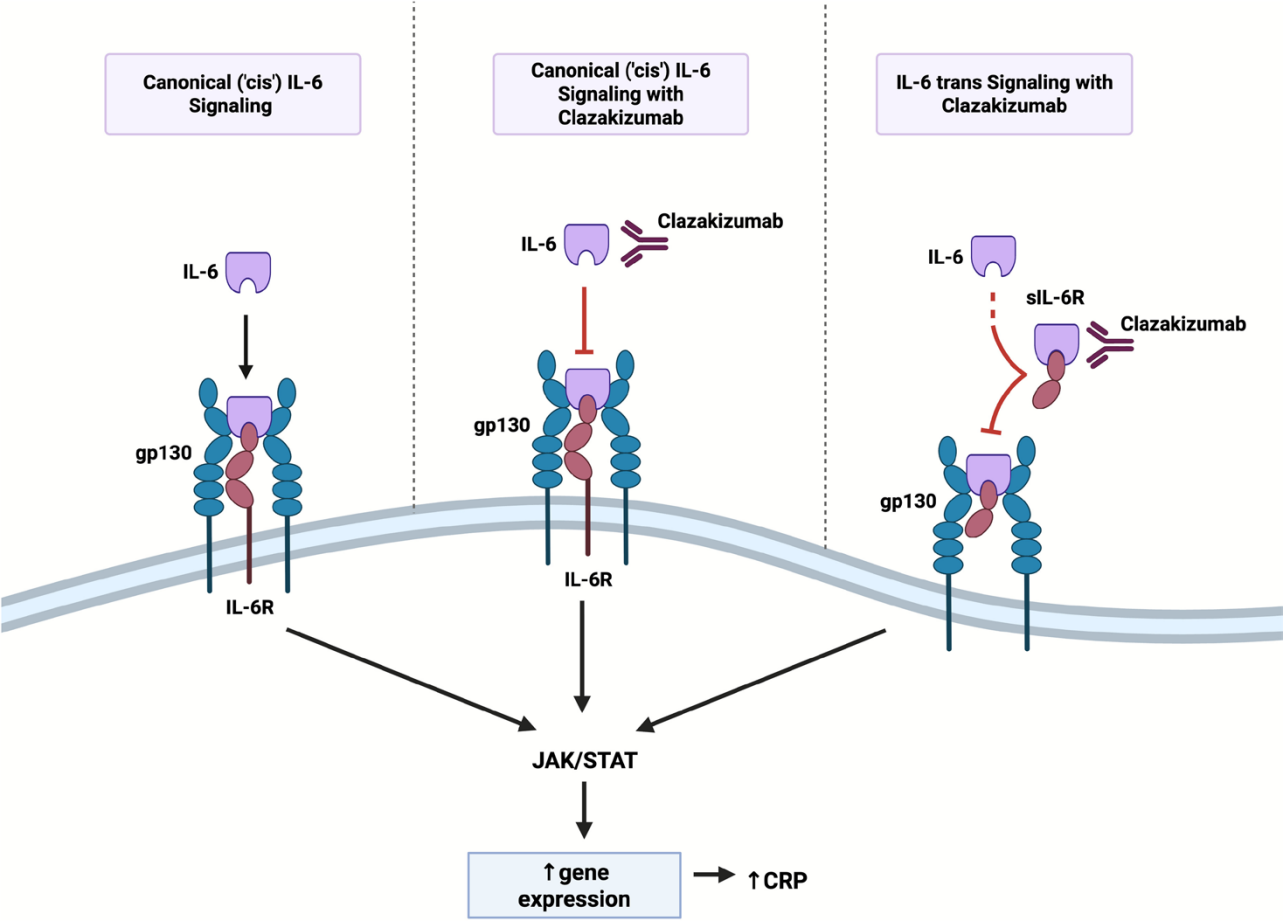
Schematic of two major modes of IL‐6 signaling. In cells that express IL‐6R, ligand binding (canonical or *cis*‐signaling) activates a number of signaling cascades including the JAK/STAT pathway, culminating in an induction of gene expression, including increased production of CRP (left panel). Cells lacking IL‐6R can also respond to IL‐6 via soluble receptor‐IL‐6 complexes (trans‐signaling) (right panel). Clazakizumab can inhibit both modes of IL‐6 signaling (middle and right panel). CRP = C‐reactive protein; IL‐6 = interleukin‐6; IL‐6R = interleukin‐6 receptor; sIL‐6R = soluble IL‐6 receptor.

Clazakizumab is a genetically engineered humanized monoclonal antibody that targets IL‐6 directly by binding to the IL‐6 ligand (Figure 3), exhibiting a strong affinity for IL‐6 and a long half‐life of approximately 30 days. It has demonstrated efficacy in treating rheumatoid arthritis (RA) [17], while showing potential benefits for psoriatic arthritis and antibody‐mediated organ rejection [23]. The safety profile of clazakizumab, established through studies using both intravenous and subcutaneous formulations, aligns with the known pharmacology of IL‐6 blockade [24, 25]. Dose‐ranging studies indicate that clazakizumab significantly reduces CRP levels at doses of 5 mg subcutaneously or higher, with most disease‐specific studies using doses between 12.5 and 25 mg/mL monthly. A recent dose‐finding study in patients on dialysis showed that a 5 mg monthly dose effectively reduced CRP levels without increasing infection risk [26]. Based on these findings, the RIGHT study utilizes a 5 mg dose every 4 weeks for 24 weeks. Clazakizumab, though supported by established pharmacology and safety data, has not yet been submitted for FDA approval for any indication and is being used in the RIGHT study under Investigational New Drug number 144315.

### 2.6 Study Drug Preparation and Dispensing

Clazakizumab is provided to the University of Pittsburgh by CSL Behring in 12.5 pg/mL vials, stored frozen at −20°C ± 5°C until the day of the injection. Syringes containing 5 mg of clazakizumab are refrigerated at 2°C–8°C (36°F–46°F) for up to 24 h, with a maximum of 4 of those hours allowed at room temperature, 15°C–25°C (59°F–77°F). Before administration, the drug is brought to room temperature over 30–60 min. Because the study drug has a slight yellow color and turbidity, both study drug and placebo syringes are covered to maintain masking and to protect from light. Study staff administer all injections to ensure safety requirements. Participants receive either clazakizumab (5 mg/month) or placebo in a one‐to‐one ratio. Participants who miss doses within the defined visit window (±10 days) remain in the study and are followed for outcome assessments to the end of the study. Dosing outside the protocol window is not permitted.

### 2.7 Study Outcomes

The primary study outcome is the change in the time to walk 400 m at a usual pace. This endpoint was chosen as a whole‐person, clinically meaningful measure of mobility commonly used as a geroscience outcome [27]. The test is conducted in a hallway accommodating a 20‐m course with testing standardized as 10 laps of 40 m and allowing up to 15 min to complete the task [28].

Secondary outcomes address four areas: additional physical function measures, brain health, markers of inflammation, and vascular function. Other physical function tests include reduction in oxygen consumption (VO_2_) with preferred and slow walking speed while wearing a portable cardiopulmonary device (Cosmed K5) [29] and perceived fatigability using the Pittsburgh Fatigability Scale [30, 31]. Brain health outcomes include cognitive tests and changes in peripheral blood biomarkers of dementia including amyloid, phosphorylated tau proteins, neurofilament light chain, and glial fibrillary acidic protein. Cognitive tests include the Digit Symbol Substitution Test, Trails B [32, 33, 34], California Verbal Learning Test (CVLT) [35], and Montreal Cognitive Assessment (MoCA) [36]. Inflammatory markers include change in serum levels of free and total IL‐6 (Rules Based Medicine Simoa IL‐6), high sensitivity CRP (hs‐CRP), and a panel of cytokines (Mesoscale V‐PLEX Plus Human Cytokine 35‐Plex) [37]. Vascular function will be assessed by standard blood pressure measures in the right arm with an automated blood pressure cuff. Aortic Pulse Wave Velocity (APWV) will be recorded using the Sphygmocor XCEL system (AtCor Medical, Sydney, Australia) [38], and endothelial function will be measured with peripheral artery tonometry (EndoPAT 2000, Itamar Medical), which calculates the reactive hyperemia index (RHI), a marker of vascular health [39].

### 2.8 Safety Parameters

Safety and tolerability of clazakizumab is assessed prior to each dose of medication and includes review of medical history and medications, review of symptoms including symptoms of active infection, and laboratory evaluation. Clazakizumab has known side effects of reducing absolute neutrophil count and/or platelet count and potential effects on liver function (i.e., transaminases, total bilirubin) and lipid levels [40], such that these will be checked monthly with well‐defined parameters for holding or discontinuing study drug. Additionally, telephone assessments are made weekly between the first two doses, 1 week after each remaining dose, and then monthly for 6 months after the final dose to identify any adverse health events or late effects of the treatment.

### 2.9 Exploratory Outcomes

In a subset of individuals meeting safety parameters, we will assess brain structure and function with a magnetic resonance imaging (MRI) scan on a 3T Siemens Prisma scanner. Brain structure will be assessed with a high‐resolution, three‐dimensional MPRAGE structural scan. Functional connectivity will be assessed with echoplanar imaging of blood oxygen level‐dependent response while resting. Structural connectivity will be assessed with a diffusion weighted scan. White matter hyperintensities will be assessed with a T2 3d FLAIR scan. Blood flow will be assessed with ASL [41].

Serum, plasma, and white blood cells will be stored at −80° for additional exploratory outcomes such as DNA methylation.

### 2.10 Statistical Considerations

#### 2.10.1 Sample Size Determination

For our primary outcome of 400‐m walking speed, changes of 0.8 m/s are considered to be meaningful. We based our power estimates on ENRGISE [20], where the 400‐m walking speed at baseline was 0.8 ± 0.2 m/s, and at 12 months the difference and standard error (SE) for the within person change of the 400‐m walking speed between losartan versus placebo was −0.025 ± 0.026 m/s and was 0.010 ± 0.014 m/s for fish oil versus placebo. After converting the SE to standard deviation (SD), these correspond to a pooled SD of 0.117 m/s. Based on budget and time allotted for this pilot project, a sample size of 60 will be used. This allows us to detect a 0.8 standardized effect size for the walking speed during a 400‐m walk under 80% power, *α* = 0.05, *σ* = 0.200 m/s and a drop‐out rate of 15% (Table S1).

#### 2.10.2 Randomization Procedure

A randomization module with a user interface that supports personal computers, laptops, tablets, and smartphones is integrated into the RIGHT web‐based data systems. The study uses a 1:1 allocation ratio using permuted block randomization with random block sizes.

#### 2.10.3 Analysis of Outcomes

Analyses will follow the intention‐to‐treat (ITT) principle. Participants who miss doses will be encouraged to stay in the study for outcome assessments. Outcomes at 6 months will be compared using non‐parametric tests (e.g., Kruskal–Wallis). If model assumptions are met, linear models will be created to assess changes from baseline in each outcome (dependent variable); treatment group (active/placebo), and the baseline value of outcome as a fixed effect covariate. The primary focus will be on mean estimates and within‐person change in order to inform a future phase III study.

Proportions of participants with AEs between the treatment groups will be compared using chi‐square or Fisher's exact tests as appropriate. Given the small sample size for this study and the rare adverse event rates, this study is unlikely to have the power to demonstrate statistical significance.

#### 2.10.4 Data Safety Monitoring

A DSMB consisting of experts in aging, mobility, rheumatology, biostatistics, and ethics meets regularly during the study at intervals determined in consultation with the DSMB. Recruitment was initiated after the DSMB approved the study protocol. Throughout the course of the trial, the DSMB reviews recruitment, retention, data quality/completeness, protocol deviations, breaches of confidentiality, AEs, serious adverse events (SAEs), unexpected problems, and incidental findings. An independent medical monitor determined the severity, relatedness, and expectedness of the SAEs. Given the pilot nature of this study, no statistical interim monitoring was required.

## 3 Discussion

The RIGHT study was designed to test the geroscience hypothesis that targeting fundamental mechanisms of biological aging, such as the hallmarks of aging, can improve healthspan and prevent morbidity [3, 42]. We selected clazakizumab, a high‐affinity humanized monoclonal antibody directly targeting IL‐6, to evaluate whether reducing inflammation can improve mobility and prevent age‐related functional decline. This study will assess other relevant outcomes such as cognition and vascular function, including the safety and tolerability of IL‐6 inhibition, while informing future, larger studies focused on targeting inflammation to modify human aging. This early‐stage, proof‐of‐concept study will provide critical preliminary data to inform the design of prospective single‐crossover trials. Importantly, the long‐term goal of this line of research is to promote healthier aging by reducing adverse consequences of chronic inflammation, thereby aiming to preserve independence and function in older adults.

Inflammaging is a hallmark of aging and a promising target for therapeutic interventions. Studies have shown that IL‐6 is independently associated with adverse outcomes in older adults [3, 42]. However, IL‐6 is a multifunctional cytokine involved in regulating immune responses, inflammation, hematopoiesis, and bone metabolism among other physiological processes. It also plays a key role in various chronic immune and inflammatory disorders such as RA and giant cell arteritis, where IL‐6 inhibitors—primarily IL‐6 receptor inhibitors—have been approved after showing disease control and improvements in health‐related quality of life [40]. Tocilizumab and sarilumab, both IL‐6 receptor inhibitors, block canonical or *cis*‐signaling (i.e., membrane‐bound IL‐6 receptors primarily on immune cells) and *trans*‐signaling (i.e., soluble IL‐6 receptor in cells lacking membrane‐bound receptors) [43]. In contrast, direct IL‐6 ligand blockade by selective inhibitors (e.g., clazakizumab, ziltivekimab) provides a broader suppression of IL‐6, potentially at lower doses of medications, which may theoretically reduce the risk of AEs. This was recently observed in trials using ziltivekimab in participants with coronary disease and clazakizumab in participants on maintenance dialysis, supporting the efficacy and safety of the dose employed in the RIGHT study [18, 26]. Given these differences, and considering the target population in aging and inflammation studies, IL‐6 ligand inhibitors may be better positioned to optimize therapeutic effects while maintaining a safer AE profile.

Previous studies have attempted to target inflammation with the goal of slowing aging or improving healthy aging [44]. Epidemiological studies showing decreased risk of colorectal cancer, cardiovascular disease, and dementia highlighted the potential interest of NSAIDs, including aspirin, as potential therapeutic options. However, no benefits were observed in clinical trials studying cardiovascular disease and sarcopenia in older adults [45, 46, 47]. Methotrexate, an immunomodulator commonly used for the treatment of RA, was proposed for the Inflammation Control for Elders (ICE, NCT02079948). This study was withdrawn though due to “potential participants having concerns about taking the study drug”, as noted on ClinicalTrials.gov [48]. Nevertheless, a recent trial studying methotrexate in adults with coronary disease did not show any benefit in cardiovascular events, possibly due to its inability to lower IL‐1β, IL‐6, or CRP [49]. In a similar way, the ENRGISE trial did not show any improvement in mobility or in reducing IL‐6 with fish oil or losartan. Other drugs, such as metformin, rapamycin, and senolytic agents, are currently being investigated for their indirect effects on inflammation [50, 51, 52]. Unlike previous approaches, we believe that directly targeting inflammation directly by inhibiting IL‐6, as proposed by the RIGHT study, is the most readily translatable approach to modify human aging.

A recent post hoc analysis of the CANTOS trial assessed the direct effect of IL‐1β inhibition with canakinumab on frailty among adults with atherosclerosis [53]. At 5‐year follow‐up, there were no differences in frailty, measured by both cumulative‐deficit frailty index and phenotypic frailty, between those randomized to canakinumab or placebo. The negative results of this study may be attributed to the insensitivity of the frailty measure or the baseline characteristics of participants, who were possibly healthier and had better risk factor control than the general older adult population. Alternatively, IL‐1 blockade might not be an optimal target for function and frailty modification. Interestingly, the RESCUE trial showed that IL‐6 with ziltivekimab led to nearly twice the reduction in hsCRP compared to IL‐1β inhibition compared in CANTOS, suggesting potential differences and favoring the effect of direct IL‐6 inhibition [18]. Considering these observations, the design of the RIGHT study will build upon and potentially answer some of the remaining questions.

The main challenge of the RIGHT study, as in ENRGISE and other similar trials, is identifying older adults who met IL‐6 criteria while having mobility impairment and were medically safe to receive study drug. This is particularly challenging given the association with multimorbidity and elevated IL‐6 levels. In ENRGISE, only 22.1% of participants who underwent a full screening visit and 5.3% of those initially screened by phone were ultimately randomized [19]. Given the nature of the intervention in RIGHT, its exclusion criteria are more stringent compared to those of ENRGISE. Another possible issue is the potential for perceived risks associated with the intervention, similar to the experience of the ICE trial. Fortunately, increasing experience with such agents seems reassuring and several safety parameters have been implemented. An important limitation of the study design is determining the optimal timing (i.e., age) of intervention, as well as other potential characteristics that identify older adults who could benefit most from the intervention in terms of effectiveness and safety. Additionally, the duration of 24 weeks could potentially limit the treatment effects. Finally, adherence in older adults is often affected by intercurrent illnesses. To address this, detailed algorithms have been developed to manage the resumption of intervention after acute illness, and all participants who remain eligible for outcome assessment will be maintained in the ITT analysis.

## 4 Conclusions

The RIGHT trial capitalizes on our previous experience on design, recruitment, and management of interventional studies in older adults. Most importantly, the use of clazakizumab will directly address the geroscience hypothesis, allowing us to better understand our ability to impact aging by targeting inflammation. This approach using IL‐6 inhibition is being successfully employed in similar contexts, such as cardiovascular disease and chronic kidney disease. While the RIGHT trial will not provide definite answers, it will represent a first step to generate critical safety and feasibility data to guide whether larger, adequately powered trials can be justified. The intent of this and other similar trials is focused on rigorously testing whether targeting hallmarks of aging can preserve function and healthier aging in older adults at risk. Promising results will require further consideration, including further discussion on access and integration with non‐pharmacologic measures to ensure equitable and responsible progress in geroscience and care of the aging population.

## Author Contributions

All authors contributed to the design of the clinical trial. S.E.S. and A.B.N. wrote the original draft of the manuscript. All authors made substantial contributions to drafting the article and revising it critically for important intellectual content and approved the final manuscript.

## Funding

The RIGHT study is supported by a grant from the WoodNext Foundation, administered by Greater Houston Community Foundation. Study drug was provided by CSL Behring, Melbourne, Australia. S.E.S. is supported by a Rheumatology Research Foundation Investigator Award and National Institute of Aging (R03AG0829283). D.E.S. is supported by R01AG060499, U19AG065188, R01AG073633, R01AG077179, and P30AG024827; Veterans Health Administration is supported by 1I21RX004409 and I01HX003518; PCORI IHS‐2021C3‐24147. T.F. is supported by P30 AG024827 and U54 AG075931. AHA SFRN ABN is supported for this work by the University of Pittsburgh Aging Institute, the Pittsburgh Claude D. Pepper Older Americans Independence Center: P30 AG024827, and the WoodNext Foundation. She has additional support from multiple NIH grants.

## Conflicts of Interest

S.E.S. reports clinical research support (clinical trials) by Amgen and GlaxoSmithKline, consulting and advisory board fees by Amgen and Sanofi (all funds paid to institution), speaker fees from Fresenius Kabi (all funds paid to the institution). S.E.S. is co‐inventor in patent application No. 63/620,471 filed by Sanofi. O.L.L. reports consulting fees from Novo Nordisk and DMSB participation for Acumen. T.F. is founder and stockholder in Generian Pharmaceuticals and Coloma Therapeutics. The other authors declare no conflicts of interest.

## Supporting information


**Data S1:** jgs70272‐sup‐0001‐supinfo.pdf.
